# Tissue Engineering for Human Urethral Reconstruction: Systematic Review of Recent Literature

**DOI:** 10.1371/journal.pone.0118653

**Published:** 2015-02-17

**Authors:** Vincent de Kemp, Petra de Graaf, Joost O. Fledderus, J. L. H. Ruud Bosch, Laetitia M. O. de Kort

**Affiliations:** 1 Department of Urology, University Medical Centre Utrecht, Utrecht, The Netherlands; 2 Department of Nephrology and Hypertension, University Medical Center Utrecht, Utrecht, The Netherlands; Michigan Technological University, UNITED STATES

## Abstract

**Background:**

Techniques to treat urethral stricture and hypospadias are restricted, as substitution of the unhealthy urethra with tissue from other origins (skin, bladder or buccal mucosa) has some limitations. Therefore, alternative sources of tissue for use in urethral reconstructions are considered, such as *ex vivo* engineered constructs.

**Purpose:**

To review recent literature on tissue engineering for human urethral reconstruction.

**Methods:**

A search was made in the PubMed and Embase databases restricted to the last 25 years and the English language.

**Results:**

A total of 45 articles were selected describing the use of tissue engineering in urethral reconstruction. The results are discussed in four groups: autologous cell cultures, matrices/scaffolds, cell-seeded scaffolds, and clinical results of urethral reconstructions using these materials. Different progenitor cells were used, isolated from either urine or adipose tissue, but slightly better results were obtained with *in vitro* expansion of urothelial cells from bladder washings, tissue biopsies from the bladder (urothelium) or the oral cavity (buccal mucosa). Compared with a synthetic scaffold, a biological scaffold has the advantage of bioactive extracellular matrix proteins on its surface. When applied clinically, a non-seeded matrix only seems suited for use as an onlay graft. When a tubularized substitution is the aim, a cell-seeded construct seems more beneficial.

**Conclusions:**

Considerable experience is available with tissue engineering of urethral tissue *in vitro*, produced with cells of different origin. Clinical and *in vivo* experiments show promising results.

## Introduction

Urethral reconstruction continues to be a challenging field for urologists. Whilst for some conditions only one or few procedures are standard, over 300 techniques are known for urethral stricture and hypospadias repair [1]. This diversity illustrates the complexity of these conditions and also indicates the lack of one perfect procedure. In addition to the surgeon’s skill, successful outcome of any procedure depends on the availability of appropriate tissue. A wide variety of tissues such as (vascularized) skin grafts, bladder and buccal mucosa have been used in urethral repair. However, all of these substitutes have limitations compared to the autologous urethral tissue, which can lead to complications (e.g. stricture formation, graft failure). Also, the amount of tissue that can be harvested from a donor site is limited; especially in the case of long defects, this could pose a problem. To overcome these problems, alternative materials for urethral reconstruction have been explored.

In the field of regenerative medicine, tissue engineering (TE) is defined as “*an interdisciplinary field that applies the principles of engineering and life sciences toward the development of biological substitutes that restore*, *maintain*, *or improve tissue function or a whole organ*” [2]. As early as the 1980s the first steps were made in culturing urothelial cells (UC) [3]. Initially, these cultures were used as an *in vitro* system to study the effects of exogenous substances on tissue. When TE started to evolve, the aim of culturing tissues changed to the replacement of damaged or absent organs. The rationale behind this latter strategy is that, with a limited amount of material (e.g. a small biopsy), a larger graft of autologous cells can be created. Since cells are autologous the problems with rejection are bypassed and, when implanted *in vivo*, the tissue possesses properties similar to those of surrounding tissue.

The native male urethra (with a length of about 18–20 cm) consists of three cell layers: the urethral epithelium, fibroblasts, and smooth muscle cells (SMC). The epithelial lining changes along the length of the urethra.

The urethra consists of three parts. The first short proximal segment is surrounded by the prostate and is called the *pars prostatica*; this is lined with urothelium of the same type as that of the bladder. The second very short segment (about 18 mm long) is called the *pars membranacea* and extends from the apex of the prostate to the bulb of the corpus spongiosum penis; it is lined with stratified or pseudostratified columnar epithelium. The third portion, called the *pars spongiosa*, has a length of about 15 cm and is also lined with stratified or pseudostratified columnar epithelium, but patches of stratified squamous epithelium are common in the pars spongiosa. Stratified squamous epithelium is also found in the terminal widened part of the canal that is surrounded by the glans penis.

The urethra is embedded in the corpus spongiosum, which is required for its blood supply. Ideally, a tissue-engineered urethra mimics the native urethra, i.e. it consists of multiple cell layers of different origin, protects the underlying tissue as an efficient barrier from urine, is vascularized, and is resistant to mechanical forces during surgery.

Several cell types or tissues are of interest for reconstruction: i) urethral epithelial lining (urothelium of [pseudo]stratified columnar epithelium) and SMC, because they form the most important layers of the urethra, ii) buccal mucosa, as this is often used as a graft in urethroplasties, iii) bladder urothelium, as this is easy to expand from small bladder biopsies or can be grown from cells isolated either from bladder washings or urine, and iv) spongious or cavernous tissue, as this surrounds the urethra. Stem cells from non-urologic tissue (such as adipose tissue) are also under investigation because they are easier to obtain and have the capacity to differentiate to urothelial and myogenic lineages.

In TE, the three main approaches for urethral repair are: reconstruction using only cultured autologous cells, reconstruction using biomaterials (called scaffolds or matrices), and a combination of both techniques using cell-seeded scaffolds. However, because cell-only constructs are often too vulnerable to be transported or handled surgically, cell-seeded scaffolds might solve these problems as they are capable of resisting mechanical forces, such as suturing. Ideally, a scaffold is biocompatible, biodegradable, bioresorbable, promotes proliferation of seeded cells and ingrowth of native cells when implanted, and has favorable mechanical and physical properties. In urethral TE two types of scaffolds are mainly used: synthetic scaffolds consisting of polymers [e.g. polyglycolic acid (PGA), poly-L-lactide-co-ε-caprolactone (PLLCL), etc.] or matrices derived from decellularized tissues such as accellular dermis, small intestine submucosa and bladder acellular matrix.

The present review aims to present a summary of recent literature on TE for urethral reconstruction. The identified studies are divided into the following categories: autologous cells, scaffold based approaches, and clinical results acquired with these techniques.

## Methods

### Literature search

**Fig 1:**
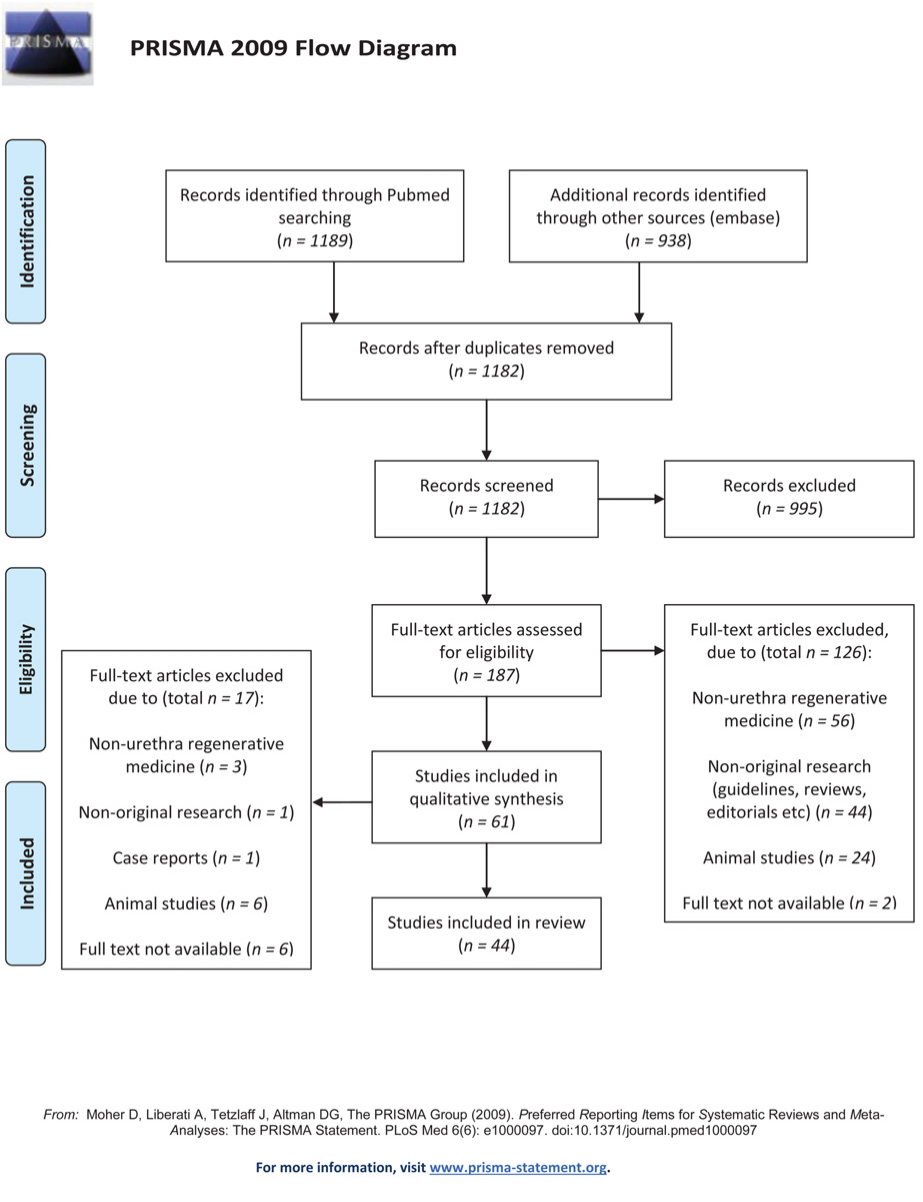
Search strategy and selection of the studies for the present review (n = 44). The database search was performed on 5 November 2013 according to the PRISMA statement [4]. For more details on the search strategy see Methods section.

A search was made (5 November 2013) in the databases of PubMed/Medline and Embase. Terms related to TE (e.g. autologous graft, stem cells, etc.) were combined with synonyms for urethra and urothelial tissue (see S1 Document). The search was restricted to the last 25 years, the English language, and studies on humans and/or human tissue. Fig. 1 presents an outline of the literature search in a Prisma Flow Diagram [4]. The Prisma Checklist [4] is included in S2 Document.

### Study selection

The results of the search were exported to RefWorks version 2.0 and duplicates were removed. Title screening was done by author VdK. Abstract screening was independently done by VdK and LdK. The two screening results were compared and any differences were resolved by discussion. Full-text screening was performed by VdK with the assistance of LdK. All studies investigating TE for urethral reconstruction (including engineering urothelial and corporal tissue) were included. Excluded were animal studies, studies on TE of (neo-)bladders or ureters, and articles in languages other than English.

### Data extraction and analysis

From the selected studies the following data were extracted. For the autologous cells section: cell type, duration of culture and authors’ conclusion. For the scaffolds and cell seeded scaffolds section: cell type, scaffold type and authors’ conclusion. For the clinical results section: number of patients, cell type, scaffold, urethral pathology, follow up and success rate. The data were entered in a database using MS office Excel 2010. As the included studies in our review vary in many parameters (e.g. cell type, scaffold choice, read out and urethral pathology), no statistical analysis could be performed.

## Results

### Autologous cells

Table 1 presents details of the 18 identified studies describing various topics related to autologous cell cultures in the context of urethral TE.

**Table 1:** Studies investigating autologous cells (n = 18).

First author (year)	Cell type	Duration of culture
Fossum (2003) [4]	Urothelium	Confluence of primary culture by day 13–21. Third generation culture obtained by day 20–28
Fossum (2005) [5]	Urothelium: Long-term culture: urothelium derived from bladder lavages of pediatric patients or short-term culture: urothelium isolated from adult urethra of gender reassignment patients. Feeder cells: immortalized mouse fibroblasts (3T3). Keratinocytes from normal donor skin	Long term culture: Cultured until senescence or until cell morphology did not reveal urothelial phenotype and absence of cytokeratins on immunoassay. Short term culture: 14 days
Nagele (2008) [6]	Urothelium acquired from bladder washings	n/a
Bharadwaj (2011) [7]	Urine-derived stem cells from upper urinary tract	n/a
Bharadwaj (2013) [8]	Urine-derived stem cells from upper urinary tract	n/a
Lang (2013) [9]	Urine-derived stem cells (USC)	3 weeks
Zhang (2008) [10]	Cells obtained from spontaneously voided or catheterized urine	n/a
Shi (2012) [11]	Adipose-derived stem cells (HADSCs)	14 days
Zhang (2013) [12]	Adipose-derived stem cells (ADSCs) and immortalized cell line from urothelium of human urinary bladder (SV-HUC-1)	2 and 4 weeks
Hudson (2007) [13]	Urothelium, surgically obtained from renal pelvis, ureter or urinary bladder	n/a
Marcovich (2003) [14]	Urothelium (UC) and smooth muscle cells (SMC)	Up to 12 days
Frimberger (2006) [15]	Cells from SDEC-cell line (human embryonic germ cells); Commercially available urothelium (URO) and bladder smooth muscle cells (SMCs)	8 days
Koskela (2009) [16]	Urothelium	n/a
Fossum (2004) [17]	Urothelial cells (UC), smooth muscle cells (SMCs) and fibroblasts (FB)	UCs grown for 1 week, seeding of FBs on the UCs, after another week SMCs seeded on top. Co-culture is incubated for 3 weeks
Cattan (2011) [18]	Dermal fibroblasts (DF) and urothelium (HUC)	14 days
Imbeault (2013) [19]	Dermal fibroblasts (DF), urothelium (UC) and umbilical vein endothelial cells (EC)	n/a
Bhargava (2004) [20]	Normal human buccal mucosa (keratinocytes and fibroblasts) on donor de-epidermized skin	Up to 14 days
Pilatz (2005) [21]	Cavernosal tissue	Primary culture, until confluent

n/a, not applicable

Because urothelial tissues are routinely harvested invasively, nine studies focused on obtaining viable cells from spontaneously voided and catheterized urine or bladder washings. Of these, three studies showed the feasibility of growing urothelial cell cultures from bladder washings, either with or without the support of feeder cells [5–7]. Four studies described the presence of stem cells in urine (USC); these USC express stem cell markers, are capable of extensive expansion, and can be induced to differentiate into all three germinal lineages [8–11]. Another possibility to acquire urological tissue is to differentiate stem cells from other tissue into the desired phenotype. Two studies demonstrated that stem cells from adipose tissue could be differentiated towards a urothelium-like phenotype [12,13].

To facilitate cell adhesion and promote proliferation, four studies investigated factors to enhance these parameters. Two studies demonstrated that immobilization of extracellular matrix (ECM) proteins on the surface of tissue culture plates resulted in enhanced cellular attachment and increased the proliferation rate of UC [14,15]. Also, increased migration rates of seeded cells were seen when they were placed under co-culture conditions with other cell types [16]. Another study showed that 17beta-estradiol induced proliferation in cultured UC [17].

Sheets composed of UC alone were too vulnerable to handle surgically. Three studies investigated the concept of a multilayered sheet consisting of different cell types to fabricate a construct strong enough for surgical handling without the need for a scaffold. Fossum *et al*. created a three-layered construct consisting of autologous urothelium, fibroblasts and SMC, using a feeder cell system [18]. In two studies from a Canadian center, a tubular graft of human fibroblasts and urothelium was created and placed under dynamic culture conditions (*e*.*g*. intra-tubular flow and pressure) that induced terminal differentiation of the urothelium [19]. An earlier vascularization of the graft was noted when this tubular graft was seeded with additional endothelial cells and implanted in the flank of athymic mice [20].

One study described the TE of a patch of buccal mucosa from a small biopsy. Oral keratinocytes were isolated from the epidermis and oral fibroblasts from the dermis and these cells were co-cultured on de-epidermized dermis (DED). The patch yielded from this co-culture was suitable for urethroplasty [21]. Another study investigated the isolation and culture of endothelial cells, SMC and fibroblasts from cavernosal tissue [22]. In the latter study, although endothelial cultures were extremely pure this could not be achieved for SMC cultures, in which fibroblasts were overwhelmingly present. The authors stated that it was difficult to discriminate, both morphologically and immunologically, between SMC and cavernosal fibroblast [22].

### Scaffold based approach

One study dealt exclusively with scaffolds [23] (Table 2). That study investigated several decellularization protocols of porcine bladders and found that one specific protocol best preserved the (bioactive) ECM proteins, thereby creating an optimal environment for tissue regeneration.

A total of 19 studies investigated cell-seeded scaffolds (Table 2). Nine studies investigated the culture of solely UC on various scaffolds. First we describe studies investigating the interaction of UC with synthetic scaffolds. Synthetic polymer scaffolds differ in the nature of the polymer, but in addition the polymers can be processed in a variety of forms, e.g. as membranes or films, and as woven, non-woven or knitted meshes. Seeding of UC onto synthetic meshes shows that, when the inter-fiber distance is large, seeded cells pass through and do not attach properly [24]. UC did not proliferate well on (non-degradable) polyethylene terephthalate (PET); however, when the surface of PET was modified, immobilized and collagen was added, and adherence and proliferation of UC was increased [25]. UC proliferated well on poly(L-lactic acid)-co-poly-(ε-caprolactone) (PLLCL) membranes without changes in the phenotype of the cells [26]. A composite scaffold of polylactic acid (PLA) with PLLCL showed less attachment and an uneven spread of UC compared to PLLCL alone [27]. Another study investigated the principle of a composite scaffold consisting of a thin poly-L-lactide (PLLA) film with electrospun polycaprolactone (PCL) on top [28]. Proliferation of seeded UC was high on the composite scaffolds compared to electrospun PCL alone. In two of these latter studies [26, 28], the proliferation of UC on synthetic scaffolds was compared with (biological) ECM scaffolds. The PLLCL scaffold was superior to human decellularized amniotic membrane, which appeared to be an unsuitable scaffold for UC [26]. The composite thin film PLLA/electrospun PCL performed better on UC proliferation and differentiation compared with SIS [28].

**Table 2:** Studies investigating scaffolds and cell-seeded scaffolds (n = 19).

First author (year)	Cell type	Scaffold type
Yang (2010) [22]	-	Porcine bladder acellular matrix (BAM)
Scriven (2001) [23]	Normal human urothelium	Hyaluronic acid derivatives (membrane/non-woven mesh); Alginate (non-woven mesh); Chitosan (non-woven mesh); Polyglactin 910 (woven mesh/knitted mesh/knitted mesh modified); PDS/Polyglactin composite (mesh); PSA/Prolene composite (mesh); Zenoderm
Bisson (2002) [24]	Normal human urothelium	Unmodified PET films; PET surfaces grafted with 0.2 or 5.9 μg/cm^2^ PAA or PET surfaces grafted with PAA and collagen or albumin (control) immobilized
Sartoneva (2010) [25]	Normal human urothelium	Human amniotic membrane (hAM) or synthetic poly-L-lactide-co-ε-caprolactone (PLCL)
Sartoneva (2012) [26]	Normal human urothelium	Different subtypes of PLCL (smooth, textured) and a composite of compression molded and knitted PLA mesh
Kundu (2011) [27]	Commercially available immortalized benign human bladder urothelial cells (TEU-2)	Composite scaffolds consisting of electrospun fibrous PCL or PLLA onto thin polymer films of PCL or PLLA compared to small intestine submucosa (SIS)
Scriven (1997) [28]	Normal human urothelium (samples from bladder, renal pelvis and ureter from 5 patients)	De-epithelialized urothelial organ culture (0.5–1.0 cm^2^), derived from biopsy during surgery for benign conditions
Sabbagh (1998) [29]	RT112, derived from a well-differentiated transitional cell carcinoma or UROtsa, an immortalized urothelial cell line	Collagen sponge, 95% type I collagen and 5% type III
Davis (2011) [30]	Commercially available normal human urothelium (HUC)	Porcine small intestine submucosa (SIS) or porcine urinary bladder matrix (UBM)
Davis (2011) [31]	Commercially available normal human urothelium (HUC)	Porcine urinary bladder matrix (UBM)
Rohman (2007) [32]	Normal human urothelium (NHU) and smooth muscle cells (SMC)	Spin-coated poly(lactide-co-glycolide) (PLGA) and poly(e-caprolactone) (PCL), both thick and thin films
Kimuli (2004) [33]	Normal human urothelium (NHU), smooth muscle cells (SM) or invasive bladder cancer cells (EJ-cell line)	Permacol, a commercially available biomaterial developed from porcine dermis by the enzymatic and chemical removal of cellular components, to leave a cross-linked collagen and elastin-rich matrix
Zhang (2000) [34]	Normal human urothelium (UCs) and smooth muscle cells (SMCs)	Commercially available small intestinal submucosa (SIS) disks, able to be seeded on both sides at once
Lakshmanan (2005) [35]	Cells from SDEC-cell line (human embryonic germ cells) subculture 7 to 9 used. Human urothelium (URO) and human bladder smooth muscle cells (SMCs)	Small intestinal submucosa (SIS)
Wu (2011) [36]	Urine-derived stem cells, differentiated in urothelial cells and smooth muscle cells or UC and SMC isolated from normal human ureter	Small intestinal submucosa (SIS)
Liu (2009) [37]	Normal human urothelium (UC) and bladder smooth muscle cells (SMC)	Porcine bladder submucosa (BSM)
Falke (2003) [38]	Normal human corpus cavernosal smooth muscle cells (SMCs) and human endothelial cells (ECs)	Collagen matrix obtained from decellularized corpus cavernosum of New Zealand White rabbits
Kershen (2002) [39]	Human corpus cavernosum smooth muscle cells (SMCs)	Non-woven sheets of polyglycolic acid polymer meshes of >95% porosity were fashioned into 22 tubular rods, 1.0 × 1.0 cm. Interfiber distances of 0–200 μm, fiber diameter 15 μm
Park (1999) [40]	Human corporal smooth muscle and endothelial cells (ECV 304)	Unwoven 1.0 × 1.0 × 0.3 cm sheets of polyglycolic acid polymer mesh, 15 μm fibers, porosity >95% before seeding
Selim (2010) [41]	Human keratinocytes and fibroblasts from buccal mucosa biopsy	Polylactide-co-glycolide (PLGA)

Four studies investigated seeding of UC on biologic materials. UC from a monolayer culture can form a stratified epithelium when suspended and re-seeded on a de-epithelialized sample of urinary tissue [29]. A sponge of collagen type I and III proved to support the growth and stratification of UC and was non-lithogenic when exposed to urine [30]. Decellularized urinary bladder matrix (UBM) proved to be significantly better than SIS regarding urothelial proliferation [31]. When seeded UBM were exposed to dynamic conditions (e.g. different pressures), urothelial proliferation was significantly improved as compared to static culture conditions [32].

In one study, UC and bladder SMC were seeded (separately) on spin-coated PLGA or PCL [33]. PLGA outperformed PCL concerning the growth of UC and SMC. It was also noted that the mechanical properties of the polymer (e.g. membrane thickness, elastic modulus) influences cell proliferation. Taken together, when UC are grown in monoculture, synthetic scaffolds and decellularized bladder matrix outperformed SIS regarding proliferation and differentiation of the cells. In addition, culture conditions and mechanical properties of the scaffold play an important role in proliferation and differentiation.

However, a more physiologically relevant situation is the co-culture of different cell types on one scaffold. Five studies investigated the co-culture of UC with SMC and/or other cells. Permacol, a decellularized porcine dermis, supported the growth of UC but not SMC when seeded alone, however, SMC did grow on Permacol when co-seeded with UC [34].

Although SIS was outperformed by UBM and synthetic polymers regarding monoculture of UC (see section above), different results were obtained in co-culture of UC with SMC. UC formed more layers and SMC better penetrated the SIS compared with SIS seeded with UC or with SMC alone [35]. Analysis of different co-culture techniques (layered, sandwich and mixed seeding) showed that the layered and sandwich technique resulted in a well-organized construct with enhanced penetration of the matrix by the SMC. Human embryonic germ cell derived stem cells also grew on SIS, but proliferation was enhanced when co-cultured with UC or SMC [36]. USC differentiated towards UC and SMC also grew well on SIS [37]. Dynamic culture conditions enhanced SMC matrix penetration of the SIS and increased the number of layers of UC and SMC. When these grafts were implanted in the flank of athymic mice, the USC-derived UC and SMC survived the implant period, and further matrix penetration as well as vascularization of the graft was seen. Similar results were obtained in a study in which UBM was used as a scaffold [38]. The decellularization process and dynamic culture influenced the proliferation rate and matrix penetration. The seeded UBM was well tolerated in the flank of athymic mice and, after one month, further proliferation and organization of seeded UC and SMC as well as graft vascularization was observed [38]. In conclusion, co-culturing of cells on scaffolds enhances proliferation and differentiation of the cell layers. Paracrine signaling between different cell types approaches the native situation. Unfortunately, the co-culture studies are all performed on biological scaffolds, which complicate comparison to synthetic scaffolds.

Buccal mucosa is studied as an additional source of epithelium for urethral reconstruction. We found one study describing the culture of human buccal keratinocytes and fibroblasts onto an electrospun PLGA scaffold [39]. Although the cells grew well on the scaffold, the sterilization process and seeding method must be well standardized as both exert considerable influence on the mechanical properties of the scaffold [39].

As the corpus spongiosum is an integral part of the urethra, TE of the corporal bodies is of interest for urethral reconstruction. Three studies described the culture of human cavernosal cells (SMC alone, or SMC combined with endothelial cells) both *in vitro* and *in vivo* (implantation in athymic mice). Two studies used synthetic scaffolds and one used decellularized corpus cavernosum of rabbits. All three studies found that the constructs formed vascularized cavernous muscle *in vivo* [40–42]. No corporal human cells other than cavernosal cells have been described in literature.

### Clinical results

Six reports were found on clinical studies in which TE techniques were used for urethral stricture repair (Table 3). Only scaffold based approaches were used, both scaffold only and cell-seeded scaffolds.

**Table 3:** Results of clinical studies using TE techniques (n = 6).

First author (year)	No. of patients	Cell type	Scaffold(s)	Urethral pathology	Follow-up (months)	Results
El-Kassaby (2003) [42]	28	n/a	Human bladder acellular matrix	Anterior strictures	36–48 (mean 37)	Successful in 24/28 patients after first attempt; successful in 4/28 patients after 1 urethrotomy
Le Roux (2005) [43]	9	n/a	Porcine small intestinal submucosa (SIS)	Bulbar strictures	12 and 24	Successful in 2/8 patients (after 12–24 months); unsuccessful in 6/8 patients (strictures within 3 months); 1 patient lost during follow-up
El-Kassaby (2008) [44]	30	n/a	Human acellular bladder matrix (ABM)	Complex anterior strictures	18–36 (mean 25)	Comparative study between repair with BM or ABM. 15/15 patients in BM group had successful outcome, 11/15 patients in ABM group had successful outcome. Poor outcome was related to previous interventions
Bhargava (2008) [45]	5	Normal human buccal mucosa (keratinocytes and fibroblasts)	Donor de-epidermized dermis (DED)	Strictures secondary to lichen sclerosis: 3 panurethral strictures, 2 panbulbar strictures	32–37 (mean 34)	Without intervention successful in 0/5 patients. 1 patient had total graft excision, 1 patient partial removal of the TEBM graft. After intervention, successful in 4/5 patients (see discussion)
Raya-Rivera (2011) [46]	5	Normal human urothelium (UC) and bladder smooth muscle cells (SMC)	Polyglycolic acid meshes (PGA)	Boys with complete posterior urethral disruption (3/5), Boys with failed previous posterior urethral repair (2/5)	36–76 (mean 64)	Successful in 4/5 patients at first attempt. One additional procedure (urethrotomy) in 1/5 patients
Fossum (2012) [47]	6	Normal human urothelium (UC) from bladder washings	Acellular dermis	Boys with scrotal or perineal hypospadia	72–103 (mean 86)	Successful in 5/6 patients at first attempt. One additional procedure (urethrotomy) in 1/6 patients. Satisfactory cosmetic appearance in 6/6 patients

n/a, not applicable

First we describe the scaffold only approach. The largest study investigated 28 men with anterior urethral strictures ranging from 1.65–16 cm in length [43]. Strictures were repaired after excision of ventral fibrotic tissue with an onlay patch of unseeded acellular collagen matrix of human cadaveric bladder. Of these 28 patients, 24 had a successful outcome, with wide caliber urethras on retrograde urethrography during follow-up. Four of the 28 had recurrent stricture at the anastomotic site and underwent endoscopic incision; hereafter, they were able to void without additional procedures. In another study, endoscopic full circumferential urethral stricture repair with unseeded SIS in nine patients had disappointing results: in six patients strictures reoccurred within three months of intervention [44]. A comparative study of 30 patients randomized and treated with standard urethroplasty with buccal mucosa graft versus urethroplasty with human acellular bladder matrix (ABM) showed good results for all buccal mucosa patients [45]. Results in the ABM group were related to the number of previous interventions: in the case of one or no previous interventions outcome was successful in 8/9 patients, with two or more previous interventions the outcome was successful in 2/5 patients; this indicates that ABM achieves the best results in patients with a healthy urethral bed (defined as no spongiofibrosis), and urethral mucosa that is fresh and vascularized. These features were mostly found in patients with one or no previous interventions [45].

In three studies cell-seeded scaffolds were used in the treatment of urethral stricture. Tissue-engineered buccal mucosa on de-epidermized dermis in five lichen sclerosis patients had mixed results, revealing problems with of the graft in 2/5 patients [46]. In one patient total graft was removed and replaced by native buccal mucosa. One patient required partial removal of the graft, one meatal dilatation, two other patients required direct vision internal urethrotomy (DVIU) within the first year. After this additional treatment, 4/5 patients still have their graft in place and a patent urethra after nine years (see discussion and [47]). In a series of five pediatric patients with posterior urethral stricture, repair with UC and SMC seeded tubular PGA scaffolds was successful in 4/5 patients [48]; 1/5 patients required transurethral incision without further interventions. The tubularized-engineered urethras showed histological and functional characteristics similar to those of native urethras, and maintained an adequate outflow for up to 6 years. In another pediatric cohort, six boys with severe hypospadias were treated with an UC seeded acellular dermis graft used in an onlay fashion [49]; 5/6 patients had a successful outcome, and in one patient an internal urethrotomy was performed after the urethroplasty. All six patients voided through their neourethras without the need for intervention.

## Discussion

This review aimed to summarize the recent literature on TE in urethral repair. During the last decade considerable advances have been made in TE and knowledge on the *in vitro* culture of tissues is rapidly expanding. However, the processes involving tissue regeneration, proliferation and differentiation are complex, comprising multiple known, as well as unknown, factors. Vascularization of the engineered graft remains a challenge and is required for successful transplantation. Cell culture techniques are well established and small amounts of cells can be expanded *in vitro*. The discovery of urinary stem cells capable of differentiation towards several cell lineages is important; this could abolish the need for invasive procedures to obtain autologous urinary tissue for *in vitro* expansion. The present study has shown that proliferation and differentiation of cultured cells are affected by both the culture conditions and type of scaffold. Ideally, the tissue-engineered graft for urethral repair should consist of different cell layers, with urethral epithelium ([pseudo]stratified columnar epithelium) at the luminal side, with an intermediate layer of SMC and a basal layer of endothelium that provides connection to the spongious tissue surrounding the urethra, as in the native urethra. Co-culturing and dynamic culture conditions enhance proliferation and differentiation of the different cell layers *in vitro*. When cells are cultured on a scaffold, a choice must be made between a synthetic and a biological scaffold. An advantage of synthetic scaffolds is that the structure and properties can be altered to specific conditions, bearing in mind that the mechanical properties of the scaffold itself influence cellular adherence and proliferation. A disadvantage of classic synthetic scaffolds is the absence of ECM proteins, which are present on biological scaffolds. These proteins are biologically active and play an important role in supporting cellular proliferation and differentiation. The decellularization process of biological scaffolds is important for preserving these ECM proteins. However, new developments include hybrid scaffolds of both synthetic and biomaterial, as well as incorporation of peptides from ECM proteins or growth factors in synthetic grafts, stimulating regeneration *in situ* rather than engineering tissue *ex vivo* [50]. Finally, 3D bioprinting, in which extracellular matrix is printed together with cell-containing hydrogels [51], will probably be a substitute for biological scaffolds in the future.

When applied in a clinical setting, the success of urethral repair with non-seeded acellular matrices correlates with the area covered by the graft. When used as an onlay graft non-seeded acellular grafts seem to work well when patients have a history of 0–2 previous interventions. After more than two interventions success rate declined [45]. Complete circumferential substitution with an unseeded scaffold was unsuccessful [44]. Both tubular substitution with a cell-seeded synthetic scaffold [48] and onlay surgery with cell seeded accellular dermis [49] showed promising results in pediatric patients; however, these results may differ in an adult study population. In a small study cohort of five lichen sclerosis patients with severe stricture disease, urethral reconstruction was performed using TE buccal mucosa (TEBM) [46]. Within the first 9 months, 2 patients had problems the graft: one patient underwent excision of the entire graft due to scarring, whereas another had partial excision due to hyperproliferation of the graft. However, in a recently published follow up [47] was reported that of the original five patients, four continue to have TEBM in situ nine years later and had a normal-looking urethra at their last follow-up, which is a good result in a lichen sclerosis patient population.

The present review has some limitations. First, the literature search specifically focused on TE of urethral and spongious tissue in order to avoid too broad a search; however, lessons can be learned from experiences with other urothelial structures and from less related areas, such as vessel TE. Secondly, to maintain the focus we excluded all animal studies, even though the results of such studies necessarily preceded investigations with human patients.

## Conclusion

Knowledge on TE in the field of urethral repair is expanding and is still finding its way into clinical implementation. Although experience with differentiation of stem cells (either isolated from urine or from adipose tissue) towards different lineages is gaining ground, protocols with *in vitro* expansion of original tissue are better established at this moment. It is noteworthy that no research has been performed with pseudostratified urethral epithelium. TE buccal mucosa has been used in urethral reconstruction, as good results have been obtained with buccal mucosa in urethral reconstruction and this tissue is easily accessible. In contrast to harvesting buccal mucosa for reconstruction surgery, TE requires only a very small of tissue, making the harvest relatively non-invasive. More invasive is the isolation of a bladder biopsy to obtain urothelium. In addition, given that ≤ 20% of the length of the urethra (prostatic urethra) is lined with urothelium and the fact that urethral reconstructions in this area are rare, urothelium would not be the first choice of tissue. Lastly, as the corpus spongiosum is often affected by fibrosis in case of urethral stricture disease, or absent in severe cases of hypospadias, a TE corpus spongiosum might enhance the success rate of a urethral reconstruction. Generating a TE corpus spongiosum together with the urethra provides protection of the urethra, healthy urethra bed and supports vascularization of the graft. TE of the corpus spongiosum has not yet been studied in a clinical setting.

Considerations regarding the choice of scaffold are that biological scaffolds have the advantage of bioactive ECM proteins on their surface and do not require a technologically challenging production process. On the other hand, bioactive synthetic scaffolds loaded with ECM components and growth factors to attract cells and provide a niche as in native tissue, are currently being tested *in vitro* and in animal models. When applied clinically, a non-seeded matrix seems suited for use only as an onlay graft. When a tubularized substitution is the aim, a cell-seeded construct seems more beneficial.

In conclusion, in view of only six reported studies (comprising 59 patients), clinical experience in this area is still relatively scarce. Moreover, due to the differing etiology of the urethral pathology in those six studies, no general conclusions can be drawn.

## Supporting Information

S1 Document Search strategy. Database search was performed on 5 November 2013 according to the PRISMA statement. More details in the Methods section of the main document.
(DOC)

S2 Document Prisma Checklist. For more information see www.prisma-statement.org.
(DOC)
