# Supplementary material for: Tissue Engineering for Human Urethral Reconstruction: Systematic Review of Recent Literature
Source: PLoS One. 2015 Feb 17;10(2):e0118653. doi: 10.1371/journal.pone.0118653 (PMC4331084; doi:10.1371/journal.pone.0118653)
Supplement: S1 Document — Database search was performed on 5 November 2013 according to the PRISMA statement. More details in the Methods section of the main document. (DOC) [file pone.0118653.s001.doc]

Supplemental Document S1

Pubmed searches

("Tissue Engineering"[Mesh]) AND ("Urethra"[Mesh] or urethr*)

102 hits

((("Tissue Engineering"[Mesh]) OR "Transplantation, Autologous"[Mesh]) OR "Stem Cells"[Mesh]) AND ("Urethra"[Mesh] or urethr*)

381 hits

((("Tissue Engineering"[Mesh]) OR "Transplantation, Autologous"[Mesh]) OR "Stem Cells"[Mesh]) AND ("Urethra"[Mesh] or urethr* or "Penis"[Mesh])

596 hits

((("Tissue Engineering"[Mesh]) OR "Transplantation, Autologous"[Mesh]) OR "Stem Cells"[Mesh]) AND ("Urethra"[Mesh] or uret* or "Penis"[Mesh])

954 hits

((("Tissue Engineering"[Mesh]) OR "Transplantation, Autologous"[Mesh]) OR "Stem Cells"[Mesh]) AND ("Urethra"[Mesh] or uret* or urethr* or "Penis"[Mesh])

1057 hits

PubMed:

((("Tissue Engineering"[Mesh]) OR autograf*[TiAb] OR autologous[TiAb] OR "tissue-engineering"[TiAb] OR "tissue engineering"[TiAb] OR "Transplantation, Autologous"[Mesh]) OR "Stem Cells"[Mesh])

AND

("Urethra"[Mesh] or uret*[TiAb] or urethr*[TiAb] or "Penis"[Mesh] OR urothel*[TiAb] OR urothelium[Mesh])

**1189 hits, limits English dutch (05-11-1988 – 05-11-2013)**

Embase:

(('tissue-engineering':ti:ab OR 'tissue engineering':ti:ab OR 'autologous transplantation') OR 'Stem Cells' OR autograft*:ti:ab OR autologous:ti:ab)

AND

(uret*:ti:ab or urethr*:ti:ab or penis:ti:ab OR urothel*:ti:ab)

**938 hits limits English (05-11-1988 – 05-11-2013)**
